# The Feeble-Minded

**Published:** 1904-10-29

**Authors:** 


### THE FEEBLE-MINDED.

### CONFERENCE AT THE GUILDHALL.

The Conference on the Feeble-minded, held recently at the Guildhall, marks an epoch not only in the history of the two societies convening it (the National Association for the Feeble-minded and the National Special Schools Union), but in that of the education of the mentally defective in this country generally.

On two points the conference asked for additional legislation, *i.e.*, with regard to special schools, and powers of detention after the age of 16; while it also sought to rouse public opinion on the matter of after-care committees in connection with the schools.

It may be noted that the use of the term feeble-minded is attended with some vagueness in the minds of many people; and, while Dr. Shuttleworth preferred to use it in the American sense, as covering the whole field of mental deficiency, Mr. C. S. Loch and others confined it to the class who are neither idiots nor imbeciles on the one hand, nor normal on the other. The "just dull"—another grade still—received their share of attention, and Dr. Eicholtz, His Majesty's Inspector of Schools, pointed out the need for dullard centres, as established in Germany, alluding also to a class on similar lines in the borough of Leicester. Much time was naturally devoted to methods of training and discipline in the schools, and an exhibition of handiwork, including some specimens from Hanover, afforded opportunities of comparison and comment; while some schools for defective children other than feeble-minded were open for visits, and excursions were arranged to some residential homes. The value of the work was abundantly testified to by teachers, inspectors, medical men, and others, and there was almost an *embarras de richesses* in the number and variety of the papers read and addresses delivered. Speech-training was dealt with by Miss Zimmerman, the difficulties of the industrial question by Miss M. K. Bradby, and the connection between mental deficiency and the maternity wards in the workhouses by Miss Douglas Tounsend, who gave an interesting account of a recently founded home for mothers and children in Shepherd's Bush. Nor was variety of views wanting, Dr. W. A. Potts, for example, maintaining that by preaching the gospel of simple food, temperance, fresh air, and hard work the problem of moral deficiency would best be solved; while other speakers doubted the correlation of deficiency and drink. On the incidental question of providing free meals, too, opinion was plainly divided, and the remark of a lady that she would rather pauperise the parents than let the children suffer received by no means unqualified support. Dr. Clemmy took the opportunity of reminding the conference of the recent deputation of medical men to the Government on the question of the teaching of hygiene in elementary schools, and a resolution in support of their view was unanimously passed. Mr. Damer Harrisson discussed mentally deficient children from a physiological standpoint, covering a wide ethnological and historic field; and Mr. Fletcher Beach laid down some guiding lines for teachers to enable them to study the varied characteristics presented by the children under their care. An interesting account of the most recent development in special schools, *i e.*, the education of cripples, was given by Miss Collard, who mentioned a class now being held for the little patients at the National Orthopædic Hospital. Some beautiful specimens of work by cripple children were on view in the hall. Dr. Wehrhahn gave an account of the education of the mentally deficient in Germany.

Mrs. Burgwin, after reading a paper on administrative difficulties in "special" work, urged that, the tentative stage being passed and the utility of these schools having been proved, it was time that the Act of 1899 should cease to be permissive, and that it should become compulsory, on the newly constituted education authorities to provide accommodation for the defective children within their respective areas. She added that the question of further provision for epileptics was being dealt with. A resolution on the lines suggested by the paper was passed.

Dr. Shuttleworth, after reviewing the history of the treatment of the mentally deficient and giving a brief account of what is being done in America and on the Continent, said there was no question that each special school should be under the ægis of an after-care committee formed of managers, teachers, and at least one interested, if not expert, medical man. They should find suitable work (as was being done in Fulham and West London) for those leaving school, permitting no interval for loafing in the streets, and exerting as far as possible a home influence; but still more important, from the sociological and scientific point of view, were the opportunities of gathering facts and figures which would throw more light upon this still obscure subject. The theory, for example, that drunkenness and heredity were chiefly accountable for mental deficiency needed proving, and it was in such a direction that he asked the committees, when formed, to exert themselves. Observations on a uniform plan should be collocated by expert persons, and opportunities should be afforded, from time to time, for discussing modifications of the plan of procedure. A specimen schedule of questions had, in accordance with this view, been drawn up by the National Association of the Feeble-minded.

Mrs. Hume Pinsent, in giving an account of after-care in Birmingham, urged, after six years' experience, that the time of such committees should be spent on the collection of scientific data rather than on attempting the almost hopeless rôle of guardian angel to individuals.

A resolution was passed to the effect that it was desirable that after-care committees should be established in connection with every special school, and that their work should be organised on broadly uniform lines.

On the question of detention it was argued by Dr. Tredgold that one-third of the entire number of mentally deficient would never be fit to stand alone, and should be, without hesitation, certified as imbeciles; and it was felt by the conference that these, to say the least, should be under permanent control in industrial homes or colonies Whether similar powers could be exerciseed in regard to those who, while capable of partial self-support, were yet a danger to the community, was not so clearly defined, and exception was taken by Miss Dendy, who has a home for boys at Liverpool, to the wage-earning capacity being adopted as a criterion. The nearer these persons approximated to the normal, she pointed out, the greater the danger. Dr. Tredgold's suggestion that some alteration was required in the marriage laws was endorsed by Dr. Milsom Rhodes, who drew attention to the stringency of the marriage laws with regard to epileptics in Massachusetts. He thought segregation could be best carried out by a combination of unions, as in Manchester and Chorlton, while Sir William Chance contended that it might be the part of the County Councils, as the education authorities, to provide accommodation. Sir Robert Anderson eloquently denounced the present policy of "quasi-philanthropic lunacy," and another speaker urged the establishment of a children's court to deal with juvenile offences, many of these being committed by the mentally deficient. The following resolution was passed—"That it should be enacted that all feeble-minded persons who, after the expiration of their training, were found to be incapable of self-support, all feeble-minded persons found to be at large without proper control or means of subsistence, or proved guilty of offences against the law, should be committed to permanent care in industrial homes or colonies"—and the need for many more such homes was urged.

